# Berberine Attenuates Axonal Transport Impairment and Axonopathy Induced by Calyculin A in N2a Cells

**DOI:** 10.1371/journal.pone.0093974

**Published:** 2014-04-08

**Authors:** Xiaofeng Liu, Jie Zhou, Morad Dirhem Naji Abid, Huanhuan Yan, Hao Huang, Limin Wan, Zuohua Feng, Juan Chen

**Affiliations:** 1 Department of Biochemistry and Molecular Biology, Tongji Medical College, Huazhong University of Science and Technology, Wuhan, China; 2 Department of Immunology, Tongji Medical College, Huazhong University of Science and Technology, Wuhan, China; New York State Institute for Basic Research, United States of America

## Abstract

Berberine is a primary component of the most functional extracts of Coptidis rhizome used in traditional Chinese medicine for centuries. Recent reports indicate that Berberine has the potential to prevent and treat Alzheimer's disease (AD). The previous studies reported that Calyculin A (CA) impaired the axonal transport in neuroblastoma-2a (N2a) cells. Berberine attenuated tau hyperphosphorylation and cytotoxicity induced by CA. Our study aimed at investigating the effects of Berberine on the axonal transport impairment induced by CA in N2a cells. The results showed that Berberine could protect the cell from CA -induced toxicity in metabolism and viability, as well as hyperphosphorylation of tau and neurofilaments (NFs). Furthermore, Berberine could reverse CA-induced axonal transport impairment significantly. Berberine also partially reversed the phosphorylation of the catalytic subunit of PP-2A at Tyrosine 307, a crucial site negatively regulating the activity of PP-2A, and reduced the levels of malondialdehyde and the activity of superoxide dismutase, markers of oxidative stress, induced by CA. The present work for the first time demonstrates that Berberine may play a role in protecting against CA-induced axonal transport impairment by modulating the activity of PP-2A and oxidative stress. Our findings also suggest that Berberine may be a potential therapeutic drug for AD.

## Introduction

The abnormally hyperphosphorylated tau and neurofilaments (NFs) are the major proteins of neurofibrillary tangles (NFT), one of the defining hallmarks of Alzheimer's disease (AD) [1], [2]. Hyperphosphorylation of tau reduces the stability of microtubules and therefore influences their organization and stability within the cell [3]. Hyperphosphorylation of tau has also been shown to mediate neurodegeneration observed in AD [4]. NFs are one of the major components of the neuronal cytoskeleton and are responsible for maintaining the caliber of axons [5]. The NFs are synthesized in cell bodies and transported into and through axons by slow axonal transport [6]. The protein subunits of NFs can be modified through enzymatic phosphorylation and dephosphorylation[7]. Several protein kinases/phosphotase are found to phosphorylate NFs and are thought to modulate NFs assembly and interact with other cytoskeleton proteins [8]–[10]. Abnormally phosphorylated NFs slow the velocity of transport of NFs and are shown to be involved in the pathogenesis of AD [11]. These data suggest that the factors contributing to the phosphorylation of tau and NFs may be critical not only to the formation of the abnormal cellular structures in the AD brains but also to the impairment of axonal transport during the pathological process of AD.

Protein phosphatase 2A (PP-2A) and protein phosphatase 1 (PP-1) are crucial phosphatases in regulating the phosphorylation of cytoskeletal proteins. Inhibition of PP-2A and PP-1 can induce AD-like hyperphosphorylation of tau and NF [12]–[16]. Previous studies show that a selective inhibition of PP-2A and PP-1 with calyculin A (CA, a specific inhibitor of PP-2A and PP-1) not only caused hyperphosphorylation of cytoskeletal proteins, but also impaired the transport of pEGFP-labeled NF-M subunit (EGFP-NFM) in the axon-like processes of N2a cells and resulted in accumulation of NF in the cell bodies [9], [17]. Thus, the impairment of the axonal transport induced by inhibition of the phosphatases may underlie the previously reported memory deficits of the rats [16], [18]. Therefore, protein phosphatases could serve as therapeutic targets for AD.

Berberine (Ber) is an isoquinoline alkaloid extracted from Chinese herbs, such as Coptidis rhizome and possesses a wide variety of biochemical and pharmacological activities. Over the past several years, pharmacodynamic studies have revealed potential roles for Berberine in the treatment of AD, including amelioration of spatial memory impairment in a rat model of AD [19]; reduction of Aβ secretion [20]; and anticholinesterase activities [21]. Previous *in vitro* study also demonstrated that Berberine attenuates tau hyperphosphorylation and cytotoxicity induced by CA [22].

Therefore, there is evidence that Berberine has protective effects on neurons damaged by CA. It would be interesting to know whether Berberine has any effects on CA-induced axonal transport impairment. Here, we have been able to express EGFP-NFM in N2a cells and to investigate whether Berberine could potentially reverse the NF axonal transport impairment induced by CA.

## Materials and Methods

### Antibodies and chemicals

Monoclonal antibodies (mAb) SMI31 and SMI32 reacting respectively to the phosphorylated and unphosphorylated NF proteins were from Sternberger Mono Inc. (Baltimore, MD, USA). The mAb tau-5 to total tau was from NeoMarkers (Fremont, CA, USA). Polyclonal antibody (pAb) PS262 to phosphorylated tau at Ser262 was from Biosource (Camarillo, CA, USA). The mAb recognizing the catalytic subunit of PP-2A (PP-2Ac) was from Upstate/Millipore (Charlottesville, VA, USA). The mAb p-PP-2A_C_ to phosphorylated PP2A_C_ at Tyr307 was from Epitomics, Inc (Burlingame, CA, USA). The mAb β-actin was from Santa Cruz Biotechnology Inc (Santa Cruz, CA, USA). Secondary antibodies for Western blotting were from Amersham Pharmacia Biotech (Little Chalfort, Buckinghamshire, UK). CA and Berberine were from Sigma Chemical Co. (St Louis, MO, USA). The bicinchoninic acid (BCA) protein detection kit, chemiluminescent substrate kit and phosphocellulose units were from Pierce Chemical Company (Rockford, IL, USA). CA and Berberine were dissolved in 1% dimethylsulfoxide (DMSO) (V/V) with a stock concentration and stored at −20°C. Dulbecco's modified Eagle's medium (DMEM), Optimem, and fetal calf serum were from Invitrogen Corporation (Gaithersburg, MD, USA). Gamma ^32^P adenosine triphosphate (γ-^32^P-ATP) was from Beijing Yahui Biologic and Medicinal Engineering Co (Beijing, China). Inhibitor-1 was from Santa Cruz Biotechnology Inc (Santa Cruz, CA, USA).

### Cell culture and outgrowth analysis of axon-like processes

Wild-type mouse neuroblastoma-2a (N2a) cell line was obtained from Dr. Xu H[23] of the Burnham Institute (La Jolla, CA, USA). We chose to use N2a cell line because it could produce large quality of microtubule proteins and grow long cell processes, and it had been highly recommended for the study of axoplasmic flow in nerve cells [24]. The cells were cultured in a 1∶1 mixture of DMEM and Optimem containing 5% fetal bovine serum (Gibco, Grand Island, NY, USA), and in a humified incubator aerated with 95% air and 5% CO_2_ at 37°C. The medium was replaced every other day, and cells were plated at an appropriate density according to each experimental scale. For the morphology studies, the cells, cultured for 24 h, were switched to serum-free medium for differentiation, and 2.5 nmol/L CA with or without 25 μg/mL Berberine was added at 12 h after serum withdrawal and the cells were cultured for another 12 h [9]. At the end of culture, cell images were taken by phase contrast microscopy and the outgrowth of the axon-like processes was analyzed using a stereological system (Stereo Investigator 2000 4.3; MicroBrightfield Inc., Williston, VT, USA). About 100–200 axon-like cell processes were counted for the quantitative analysis.

### Measurement of cell viability and metabolism

The cells were seeded onto 96-well culture plates at density of 3×10^5^ cells/well in 100 μL. For CA or Berberine treatment, different concentrations of CA (2.5, 5.0, 10 nmol/L) or Berberine (5, 10, 25, 50, 100 μg/mL) or culture medium containing 0.01% of DMSO, used as a vehicle control, were added to the culture medium and incubated with the cells for 12 hr. Crystal violet and 3-[4,5dimethylthiazol-2-yl]-2,5-diphenyl-tetrazolium bromide (MTT) assays [25] were used to detect cell viability and metabolism. MTT, dissolved in phosphate-buffered saline (PBS) at 5 mg/mL, was added to the cells and incubated for 6 hr at 37°C, and the cells were lysed with DMSO before being read at 570 nm (Tecan, Salzburg, Austria). For crystal violet assay, 0.2% crystal violet in PBS was added and incubated with cells for 2–3 min, then 1% SDS was added and the color was read at 570 nm. All assays were performed in octuple repeats.

### Live-cell imaging and image analysis

pEGFP-NFM was transiently transfected into N2a cells using Lipofectamine 2000, according to the manufacturer's instruction (Invitrogen, Carlsbad, CA, USA). These cells were plated for about 20 to 24 h and then transfected with pEGFP-NFM expression plasmids. The cells used for axonal transport study were cultured without serum for 12 h before addition of CA with or without Berberine. After a 12 h treatment with 2.5 nmol/L CA with or without 25 μg/mL Berberine in serum-free medium, cells were then observed by evanescent-field (EF) fluorescence [9], [10]. Exposure time (100–300 ms) and interval time (4–7 s) were adjusted according to the fluorescence intensity of images. Single and time-lapsed images were viewed, processed and analyzed by TILL vision 4.0 and Photoshop 7.0. Image data were analyzed using Igor Pro 4.03 (Wave Metrics Inc., Oregon, USA). For these analyses, we only selected filaments that were undergoing bouts of sustained unidirectional movement. Approximately 90–100 vesicles in approximately 20 cells per sample were counted for the axonal transport speed study.

### PP-2A activity assay

Phosphorylase-b (2 mg/mL) was phosphorylated into phosphorylase-a by incubating it for 10 min at 30°C in 40 mmol/L Tris–HCl pH 8.5, 20 mmol/L β-ME, 0.2 mmol/L CaCl_2_, 15 mmol/L MgCl_2_, 0.5 mmol/L γ-^32^P-ATP, and 10 mg/mL phosphorylase kinase. The product of γ-^32^P-phosphorylase was separated from free ATP on a Sephadex G-50 column, and the protein-containing fractions were collected. The activity of PP-2A toward γ-^32^P-phosphorylase-a was assayed by the release of γ-^32^P as described previously [9]. The reaction was carried out in a 20 mL reaction mixture containing 50 mmol/L Tris, pH 7.0, 10 mmol/L β-ME, 0.1 mmol/L EDTA, 7.5 mmol/L caffeine, 7.5 ng/mL γ-^32^P-phosphorylase-a, 0.2 mg/mL inhibitor-1 (PP-1-specific inhibitor), and 0.06 mg/mL cell lysates. The reaction was started by adding γ-^32^P-phosphorylase-a, after incubation for 30 min at 30°C, the reaction mixture (5 μL) was spotted on a chromatography paper already spotted with 10 μL stop solution (4 mmol/L cold ATP in 20% trichloroacetic acid). The released γ-^32^P was separated from the substrate by ascending chromatography in 5% trichloroacetic acid (in 0.2 mol/L NaCl), and the radioactivity was counted by liquid scintillation counting.

### Western blotting

Cell cultures were rinsed twice in phosphate buffered saline and then lysed in sample buffer containing 50 mmol/L Tris–HCl pH 8.0, 150 mmol/L sodium chloride, 1% NP-40, 0.5% sodium deoxychlolate, 0.1% sodium dodecyl sulfate, 0.02% sodium azide, 100 μg/mL phenylmethysulfonyl fluoride, 1 μg/mL aprotinin, spun at 12 000 g for 20 min at 4°C. The supernatants were mixed immediately by adding sample buffer. Protein concentration was determined with BCA Reagent. Equal amounts of protein (30 μg) were isolated in 7.5% (for NF proteins) or 10% sodium dodecyl sulfate-polyacrylamide gel, and blotted onto polyvinylidene difluoride membranes (PVDF; Amersham Pharmacia Biotech, Piscataway, NJ, USA). The membranes were blocked with 5% bovine serum albumin in Tris-buffered saline for 1 h at ∼25°C, and then probed with primary antibodies SMI31 (1∶5000), SMI32 (1∶5000), tau-5 (1∶500), PS262 (1∶1000), β-actin (1∶10000), p-PP-2Ac (1∶5000) and PP-2Ac (1∶2000). The blots were developed with horseradish peroxidase-conjugated secondary antibody and visualized by enhanced chemiluminescence substrate system (Santa Cruz, CA, USA). Quantification of protein bands was performed via scanning with the Bio-Rad GelDoc™ XR and Chemi Doc™ XRS systems and the bands were analyzed by Quantity One 1-D Analysis Software Version 4.6.3.

### Measurement of malondialdehyde and superoxide dismutase

Cells were seeded onto 6-well culture plates, after exposure to CA for 12 h with or without Berberine treatment, then cells were washed twice with phosphate-buffered saline (PBS) (pH 7.4, at 4°C) and lysed with buffer containing 10 mM Tris–HCl (pH 7.4), 10 mM ethylenediaminetetra–acetic acid, 0.2% Triton X-100 for at least 15 min on ice. The cells were scraped from the wells and centrifuged at 17000 *g* for 15 min at 4°C. Superoxide dismutase activity in the supernatant was determined by assessing the inhibition of pyrogallol autooxidation [26]. Malondialdehyde (MDA), a metabolite of lipid peroxides, was used as an indicator of lipid peroxidation. Plasma MDA concentrations were estimated as reactive substances by thiobarbituric acid adduction [27]. In brief, N2a cells were washed twice and collected with cold PBS, centrifuged at 3000 *g* for 5 min, and then the pellets were mixed with 4 ml of 1/12 M sulfuric acid and 0.5 ml of 10% phosphotungstic acid thoroughly. After centrifugation at 3000 *g* for 10 min, the liquid phase was decanted. Four milliliter double-distilled water and thiobarbituric acid reagent (0.67% 2-thiobarbituric acid/acetic acid 1∶1) were then added to each sample, mixed and heated in water bath at 95°C for 1 h. Samples were cooled with tap water and 5 ml *n*-butanol–alcohol was added, and the samples were vigorously shacked for 1 min and centrifuged. The *n*-butanol–alcohol phase, which contains the lipid peroxides, was used for MDA analysis with fluorospectrophotometer (F-2000, Hitachi Ltd. Tokyo, Japan) at excitation/emission of 515/553 nm. Freshly diluted tetrametoxypropane, which yields MDA, was used as a standard and results are expressed as nanomoles of MDA equivalents. Protein concentration was measured with BCA Reagent (Pierce, Florida, USA).

### Statistical analysis

Data were expressed as mean ± SD and analyzed using spss 10.0 statistical software (SPSS Inc., Chicago, IL, USA). The one-way anova procedure followed by least significant differences *post hoc* tests was used to determine the different means among groups.

## Results

### Effects of Berberine on cell metabolism and cell viability

**Figure 1:**
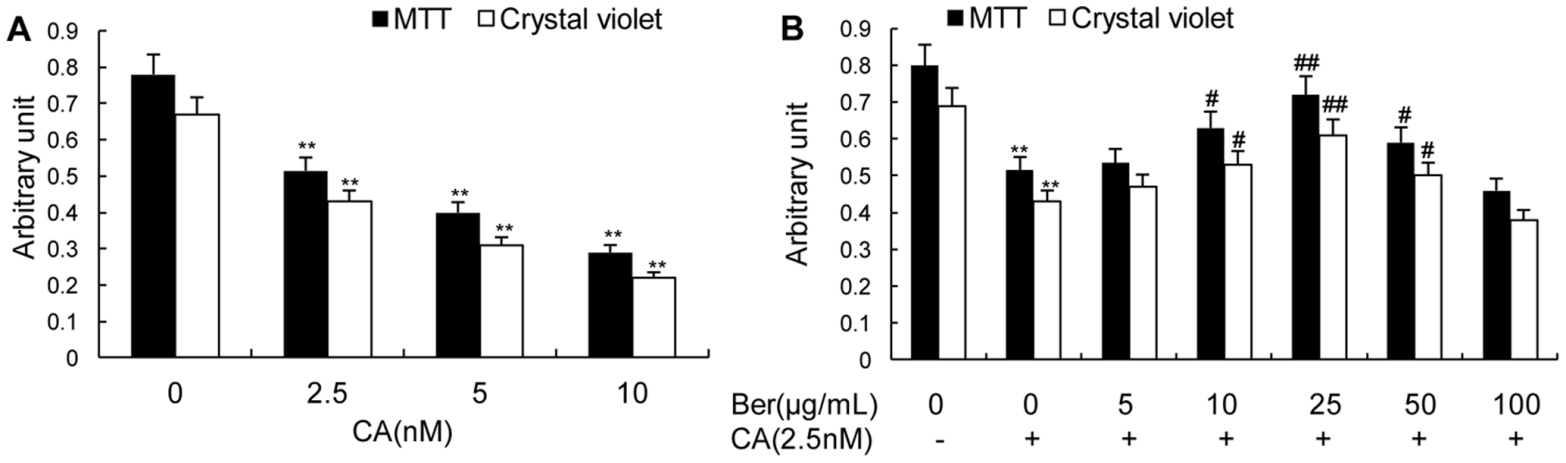
CA-induced MTT and crystal violet reduction and protection by Ber in N2a cells. CA decreased cell viability in a dose-dependent manner as determined by MTT and crystal violet assay (**A**) and Ber increased cell viability in a dose-dependent manner until to 25 μg/ml in 2.5 nmol/L CA-treated cells (**B**). 25 μg/ml Ber showed significant protection of the cells from CA-induced lesions. When the concentration of Ber was higher than 50 μg/mL, the neuroprotective effects were weakened instead. Data are mean ± SD (n = 6). ^*^P<0.05 or ^**^P<0.01 versus vehicle control. ^#^P<0.05 or ^##^P<0.01 versus CA-treated cells.

CA was a specific inhibitor of PP-1 and PP-2A. Previously report showed that 2.5 nmol/L CA not only caused hyperphosphorylation of cytoskeletal proteins, but also impaired the transport of pEGFP-NF-M in the axon-like processes of N2a cells [17]. In this study, we also found that CA decreased cell metabolism and cell viability in a dose-dependent manner as determined by MTT and crystal violet assay (Fig. 1A). Although greater inhibition of PP-2A was seen by using 5.0 nmol/L of CA, the cell morphology at this condition was not good enough for the axonal transport analysis in live cells (data not shown). Therefore, we chose 2.5 nmol/L of CA for the rest of our experiments. Berberine could increase cell metabolism and cell viability in a dose-dependent manner until to 25 μg/mL. When the concentration of Berberine was higher than 50 μg/mL, the neuroprotective effects were weakened instead (Fig. 1B). Therefore, we chose 25 μg/mL of Berberine for our study.

### Effects of Berberine on PP-2A activity

**Figure 2:**
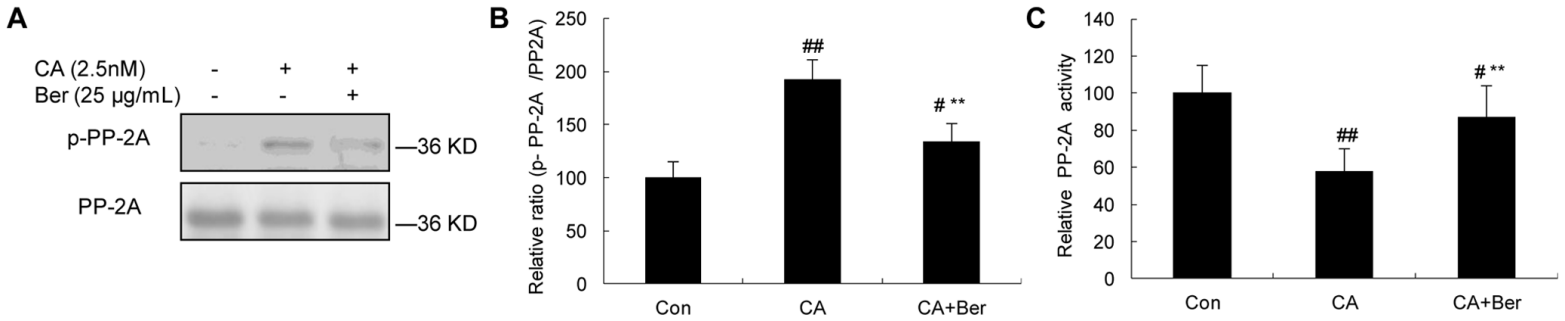
The effect of Ber on the phosphorylation and activity of PP-2A in N2a cells, induced by CA. The phosphorylation of PP-2Ac (Y307) was significantly increased in CA-treated cells compared with that of controls. Ber (25 μg/mL) reversed the phosphorylation of PP-2Ac (**A**). Quantitative analysis showed the relative ratio of p-PP-2Ac to PP-2Ac (**B**). The activity of PP-2A was measured by a ^32^P-labeling assay (**C**). Ber (25 μg/mL) significantly reversed the activity of PP-2Ac. ^#^p<0.05 and ^##^p<0.01 versus vehicle control; ^**^p<0.01 versus CA-treated cells (n = 4).

The inhibition of PP-2A was shown by an increased immunoreactivity of p-PP-2Ac that recognizes the phosphorylated (i.e., inactivated) PP-2Ac at Tyr307. The mAb PP-2Ac to total PP-2Ac was used to normalize the phosphorylated level of PP-2Ac (Fig. 2A and B). By using a ^32^P-labeling assay, we observed that the activity of PP-2A was reduced by ∼32% after treatment with 2.5 nmol/L CA for 12 h (Fig. 2C). However, treatment of the cells with 25 μg/mL Berberine antagonized this effect by 13%. Berberine could significantly restore the activity of PP-2A.

### Effects of Berberine on tau and NF phosphorylation

**Figure 3:**
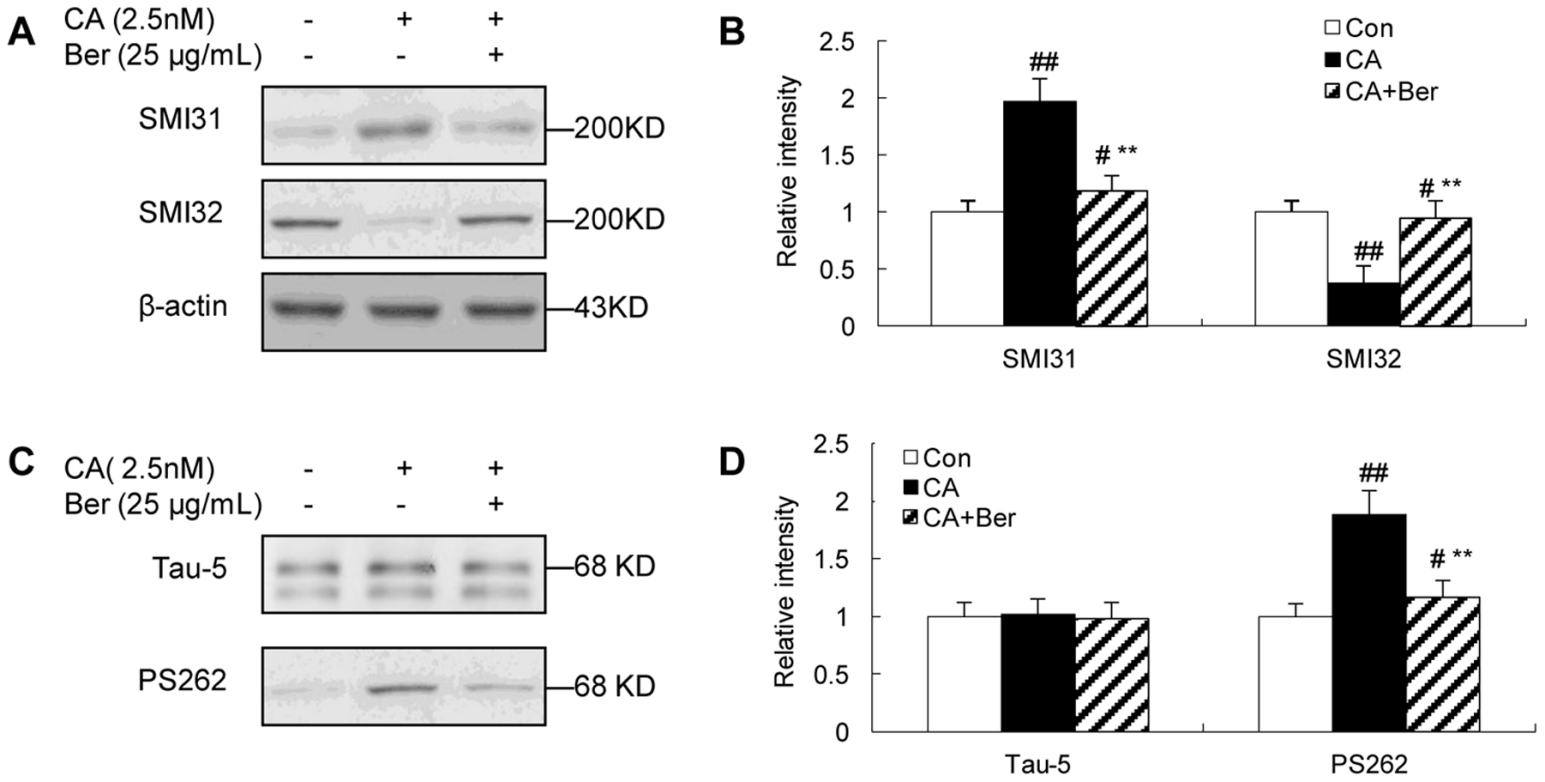
The effect of Ber on the phosphorylation of tau and NFs in N2a cells, induced by CA. Western blots of cell lysates treated with CA (2.5 nmol/L) and/or with Ber (25 μg/mL) for 12 h and the blots were developed with phosphorylation-dependent tau antibody (PS262) and NF antibody (SMI31). Increased phosphorylation of NF-H/M probed by SMI31 and SMI32 was observed (**A and B**). The phosphorylation level of tau was normalized against total tau probed by Tau-5. The phosphorylation of tau was significantly increased at Ser262 epitope in CA-treated N2a cells (**C and D**). Supplement of Ber (25 μg/mL) arrested hyperphosphorylation of tau and NFs induced by CA. β-actin was used as loading control. ^#^p<0.05 and ^##^p<0.01 versus vehicle control; ^**^p<0.01 versus CA-treated cells (n = 4).

To determine whether CA affected the phosphorylation of neuronal cytoskeletal proteins and the effect of Berberine, Western blot analysis was performed using antibodies specifically recognizing the phosphorylated or nonphosphorylated NF. It was shown that 2.5 nmol/L of CA treatment for 12 h obviously increased the immunoreaction of phosphorylated NF recognized by SMI31 (Fig. 3A and B), while 25 μg/mL Berberine significantly attenuated the hyperphosphorylation. On the contrary, CA depressed the immunostaining of nonphosphorylated NF detected by SMI32 (Fig. 3A and B) and Berberine partially inhibited the decrease. Additionally, CA treatment resulted in an increased immunoreaction of the phosphorylated-tau at Ser262 (Fig. 3C and D), a crucial site for the microtubule-binding activity of tau while 25 μg/mL Berberine also significantly attenuated the hyperphosphorylation of tau. These results confirmed in N2a cells that inhibition of the phosphatases by CA induced hyperphosphorylation of NF and tau proteins and Berberine partially attenuated the hyperphosphorylation.

### Effects of Berberine on NF axonal transport

**Figure 4:**
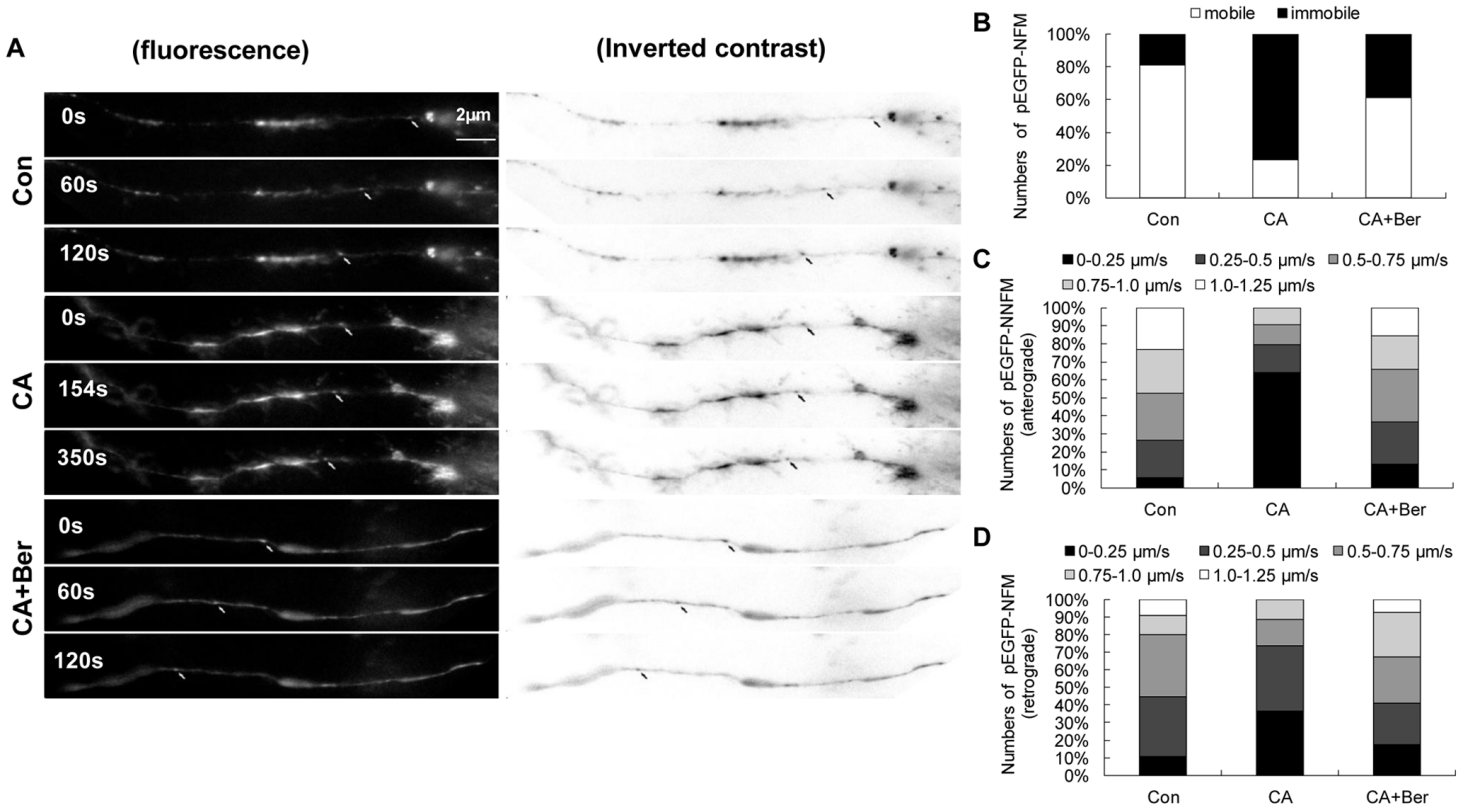
Ber protects against CA-induced slowing of NF axonal transport in N2a cells. The pEGFP-NFM was transfected into N2a cells for 4 h, and then the culture medium was replaced by serum withdrawal for 12 h, followed by treatment with vehicle or CA (2.5 nmol/L) in either the presence or absence of Ber (25 μg/mL) in serum-free medium for 12 h. The time-lapse images for the transport of pEGFP-NFM were recorded and the velocity of the movement was calculated as described in the method portion of the context. For these analyses, we only selected filaments that were undergoing bouts of sustained unidirectional movement. (**A**) A forward-moving pEGFP-NFM particle in control and a relatively stationary particle in CA-treated cell process. Ber reversed CA-induced slowing of NF axonal transport significantly. (**B–D**) Quantitative analyses show that the proportion of the cells with immobile and slow-moving pEGFP-NFM increased significantly after CA treatment and this was rescued by treatment with Ber (25 μg/mL). About 90-100 vesicles in ∼20 cells for each treatment were counted. Bar = 2 μm.

Previous report showed that CA not only caused hyperphosphorylation of cytoskeletal proteins, but also impaired the axonal transport in N2a cells [9], [17]. Following the observation of NF and tau proteins phosphorylation in cultured N2a cells, experiments were conducted to explore the impact of Berberine on the impairment of axonal transport induced by CA. The cultured N2a cells were transiently transfected with pEGFP-NFM, which enabled the direct observation of NF axonal transport by live fluorescence imaging. When N2a cells were transfected with pEGFP-NFM for 24 h, numerous fluorescence particles were seen to move along axons. The majority of the particles were moving at an average velocity of about 1 μm/s in an anterograde direction, while only a few particles moved at a slower speed of 0.26 μm/s in a retrograde direction (average velocity). Very few immobile fluorescence particles were observed (Fig.4).

To determine whether Berberine influences CA-induced slowing of NF transport, we treated neurons with CA in either the presence or absence of Berberine and monitored NF movement through axons. The treatment of cells with 2.5 nmol/L CA induced a decrease by 77% in the velocity of EGFP–NFM. However, treatment of the cells with 25 μg/mL Berberine antagonized this effect by 28.9%. It is important to note that the treatment with Berberine alone showed no effect on transport (Data not shown), indicating that the effect of Berberine was CA-related. These data indicated that the treatment of the N2a cells with CA slowed the velocity of EGFP-NFM transport and Berberine reversed CA-induced axonal transport impairment significantly (Fig.4).

### Effects of Berberine on cell morphology

**Figure 5:**
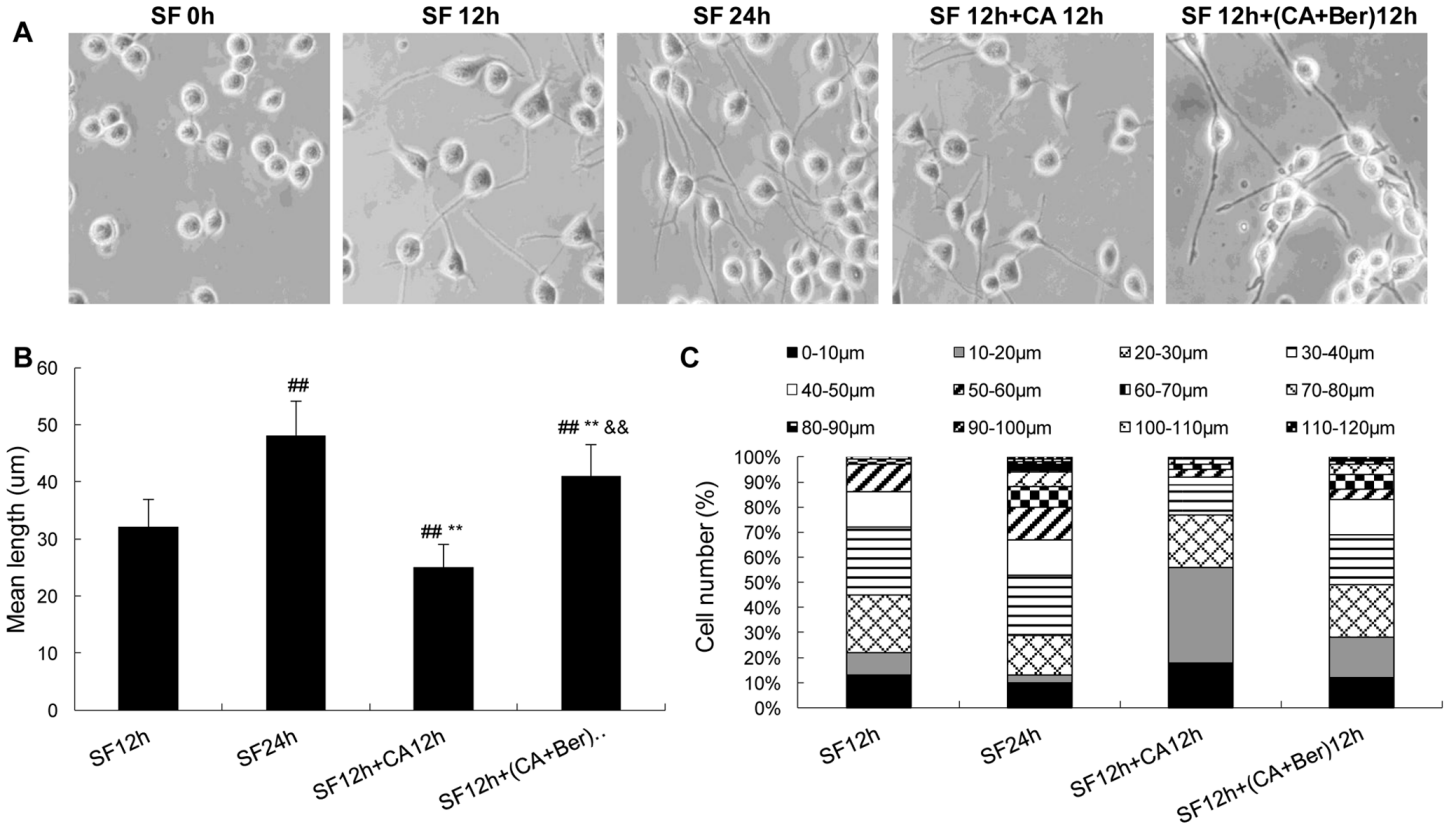
CA inhibits serum withdrawal-induced outgrowth of axon-like cell processes and protection by Ber in N2a cells. The cells were cultured in serum-free (SF) medium for 12 or 24 h, then 2.5 nmol/L CA was added to the 12 h SF group and these cells were cultured for another 12 h. The morphology of the cells was photographed by a phase contrast microscope (**A**). The mean length of the cell processes (**B**, excluding those with zero length of the processes) and the proportion of cells with different lengths of the processes (**C**) were calculated by a stereological system. About 100–200 axon-like cell processes for each treatment were counted. Data were mean ± SD (n = 5). ^##^p<0.01 versus SF 12 h, ^**^p<0.01 versus SF 24 h, ^&&^p<0.01 versus SF 12 h+ CA 12 h. Bar  = 10 μm.

The effect of Berberine on cell morphology was studied by a phase-contrast microscope and a stereological analysis system. To make this analysis possible, we established a cell model showing stable outgrowth of the axon-like cell processes by serum withdrawal [9]. We observed that serum withdrawal stimulated outgrowth of the cell processes time-dependently within 24 h (Fig 5). When CA was added to the culture at 12 h of the serum withdrawal, it was shown that CA not only inhibited serum withdrawal-induced outgrowth but also led to retraction of the already formed cell processes (Fig 5). When the cells were treated simultaneously with 2.5 nmol/L CA and 25 μg/mL of Berberine, the inhibition of axonal outgrowth induced by CA was almost restored to the normal situation. The quantitative data also revealed that the proportion of the cells with processes shorter than 20 μm was decreasing during serum withdrawal, but this value was increased by treatment of CA (Fig 5). Additionally, ∼19% of the cells had the processes longer than 60 μm at 24 h of the serum withdrawal but this type of long process was very rarely seen in CA-treated cells (Fig 5). When the cells were treated simultaneously with 2.5 nmol/L CA and 25 μg/mL Berberine (Fig. 5), the proportion of the cells with the processes longer than 60 μm was increased to ∼13%. These data indicated that CA inhibited serum withdrawal-induced outgrowth of axon-like cell processes and caused degenerative morphologies in N2a cells while Berberine partially reversed this effect (Fig.5).

### Effects of Berberine on oxidative stress

According to the previous report that CA induces oxidative stress, and Berberine prevents oxidative damage [22], [28], [29], we propose that the antioxidative effect of Berberine may contribute to its improvement of the axonal transport impairment induced by CA.

**Figure 6:**
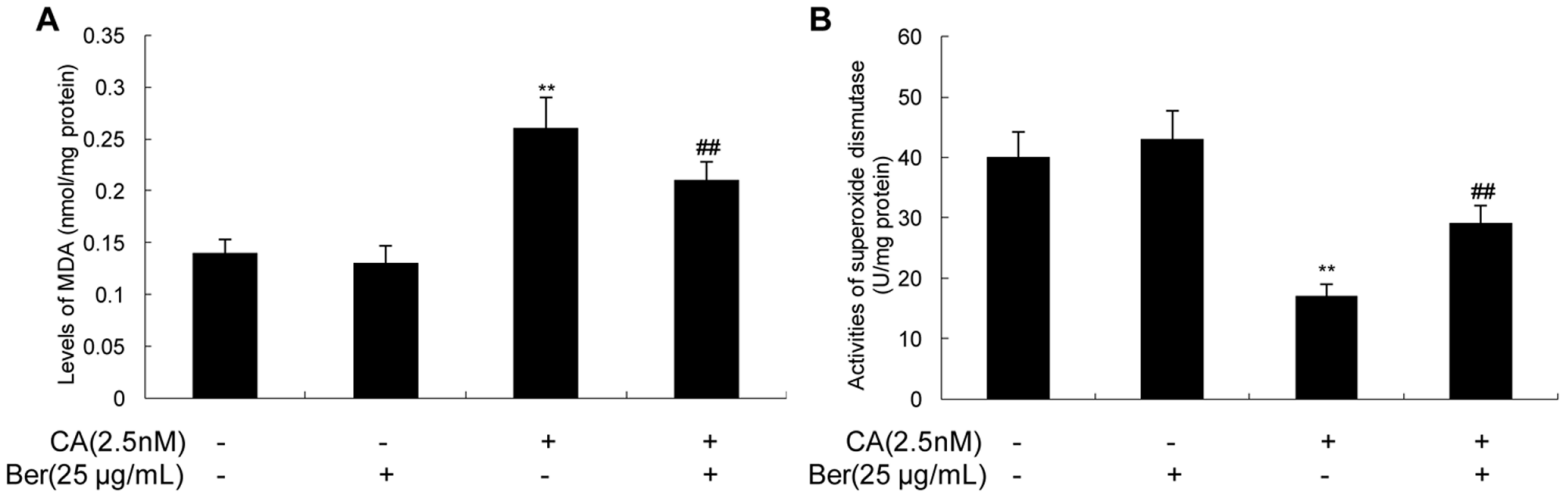
Effect of Ber on CA-induced oxidative stress in N2a cells. The levels of MDA (**A**) and the activities of superoxide dismutase (**B**) of N2a cells after 12 h exposure to CA (2.5 nmol/L) with or without Ber (25 μg/mL). Ber with concentrations of 25 μg/mL significantly blocks CA-induced elevation of MDA and rescues the activity of superoxide dismutase. ^**^ *P*<0.01 vs. vehicle control, ^##^ *P*<0.01 vs. CA-treated cells.

We measured the level of MDA as indicator of lipid peroxidation and activity of superoxide dismutase. We found that incubation of N2a cells with CA resulted in oxidative stress, characterized by a significant increased level of MDA and a decreased activity of superoxide dismutase. When the cells were treated simultaneously with CA and Berberine, the level of MDA obviously decreased (Fig. 6), and the activity of superoxide dismutase significantly increased compared with CA group (Fig. 6). No significant change was seen in the MDA level and superoxide dismutase activity by treatment of the cells with Berberine alone at 25 μg/mL.

## Discussion

Hyperphosphorylation of cytoskeletal proteins plays an important role in the formation of NFTs in AD. Abnormally phosphorylated NFs slow the velocity of transport of NFs [10], [30] and lead to the axonal transport impairment [31]–[33]. Additionally, microtubule serves as a track for the axonal transport and hyperphosphorylation of tau disrupts microtubules. The damaged axonal transport is involved in the progression of AD [11], [34] and this deficit is proposed to be diagnostic and therapeutic target for early AD [35].

Previous *in vitro* and *in vivo* studies reported that PP-2A is the most effective phosphatase for the dephosphorylation of hyperphosphorylated tau and NFs [9], [18], [36], [37]. CA is a selective inhibitor of serine/threonine protein phosphatase of PP-2A and PP-1 classes, and it has little or no direct effect on other phosphatases or kinases [38], [39]. Our present results show that CA inhibits PP-2A activity, increases phosphorylation of tau and NFs, and slows the velocity of transport of NFs resulting in the accumulations of NFs, consistent with some other investigations [10], [40].

Previous studies have also reported that Berberine arrests tau hyperphosphorylation induced by CA [25], [41], [42]. However, it is not known whether Berberine may also exert the effects on axonal transport impairment caused by CA inhibition of PP-2A. Therefore, we investigated the potential role of Berberine in regulating the process of the axonal transport in N2a cells.

Our observations demonstrated that Berberine could modulate the activity of PP-2A in the presence of CA, as evidenced by the decreased phosphorylation of PP-2Ac at Y307 site.

It was found in the present study that Berberine efficiently attenuated CA-induced hyperphosphorylation of NFs and it dephosphorylated tau at Ser262, which was proposed to be the most critical site for the stability of microtubules [43]. PP-2A is a major protein phosphatase involved in tau and NFs phoshporylation in AD [44],[45]. The phosphorylation of catalytic subunit of PP-2A (Y307) has been shown to negatively regulate the activity of PP-2A. Berberine has been shown to have a direct regulatory effect on the activities of protein kinases and protein phosphatases [22]. Here, we demonstrated for the first time that Berberine significantly improved the axonal transport impairment and alleviated ‘drawing back’ and outgrowth inhibition of axon-like processes by CA treatment in differentiated N2a cells. However, single Berberine treatment had no significant effect on the phosphorylation of NFs and tau (data not shown). The data indicated that Berberine had protective role when the neuronal cells were threatened by the inhibitory effects of CA on PP-2A and PP-1. The susceptibility of phosphorylated NF-H and NF-M to the lipid peroxidation product trans-4-hydroxy-2-nonenal is higher than that of the dephosphorylated form [46]. The neurites are more sensitive to oxidative stress due to the exclusion of mitochondria from the cell processes, implying a local depletion of ATP, causing impairment of axonal transport, destruction of the cell processes and eventually speeding up degeneration. A great deal of evidence shows that CA induces oxidative stress, and Berberine prevents oxidative damage [47]. Our data demonstrated that the antioxidative effect of Berberine might contribute to its improvement of the axonal transport impairment induced by CA in N2a cells.

In an attempt to explore the underlying causes for the impairment of the axonal transport and the reverse of the impairment, we measured MDA level and superoxide dismutase activity. Our data showed that Berberine also partially reversed CA-induced MDA elevation and CA-suppressed superoxide dismutase activity, indicating that Berberine could modulate CA-induced oxidative stress. Moreover, we have also demonstrated that vitamin E, a recognized antioxidant, did not affect the activity of PP-2A and the axonal transport impairment induced by CA (data not shown). This means that Berberine has effects that are not replaced by the general antioxidants and the mechanism by which it partially abolished CA-induced oxidative stress is specifically elaborated needs further investigation.

Based on these data, it is reasonable to believe that Berberine may be important in maintaining the physiological activity of PP-2A as well as in the regulation of the axonal transport. Our data provided evidence that Berberine might be neuroprotective in preventing the axonal transport impairment induced by CA via modulating the activity of PP-2A and oxidative stress. The precise molecular mechanisms leading to the modulation effect of Berberine on PP-2A activity need further clarification in future studies.

In conclusion, we have found that Berberine modulated the activity of PP-2A as well as oxidative stress, and reversed hyperphosphorylation of tau and NFs, the axonal transport impairment induced by CA in N2a cells.
