# Correction: Berberine Attenuates Axonal Transport Impairment and Axonopathy Induced by Calyculin A in N2a Cells

**DOI:** 10.1371/journal.pone.0152609

**Published:** 2016-03-24

**Authors:** Xiaofeng Liu, Jie Zhou, Morad Dirhem Naji Abid, Huanhuan Yan, Hao Huang, Limin Wan, Zuohua Feng, Juan Chen

The authors would like to correct Fig 5, as errors were introduced in preparation of this figure for publication. In panel A, the SF 12h panel and the SF 12h+CA 12h panel are erroneously derived from the same image. The authors have provided a corrected version of Fig 5 here that includes a new image for the SF 12h+CA 12h panel. The raw images used to create the revised panel can be viewed as Supporting Information. The authors confirm that this change does not alter their findings.

**Fig 5:**
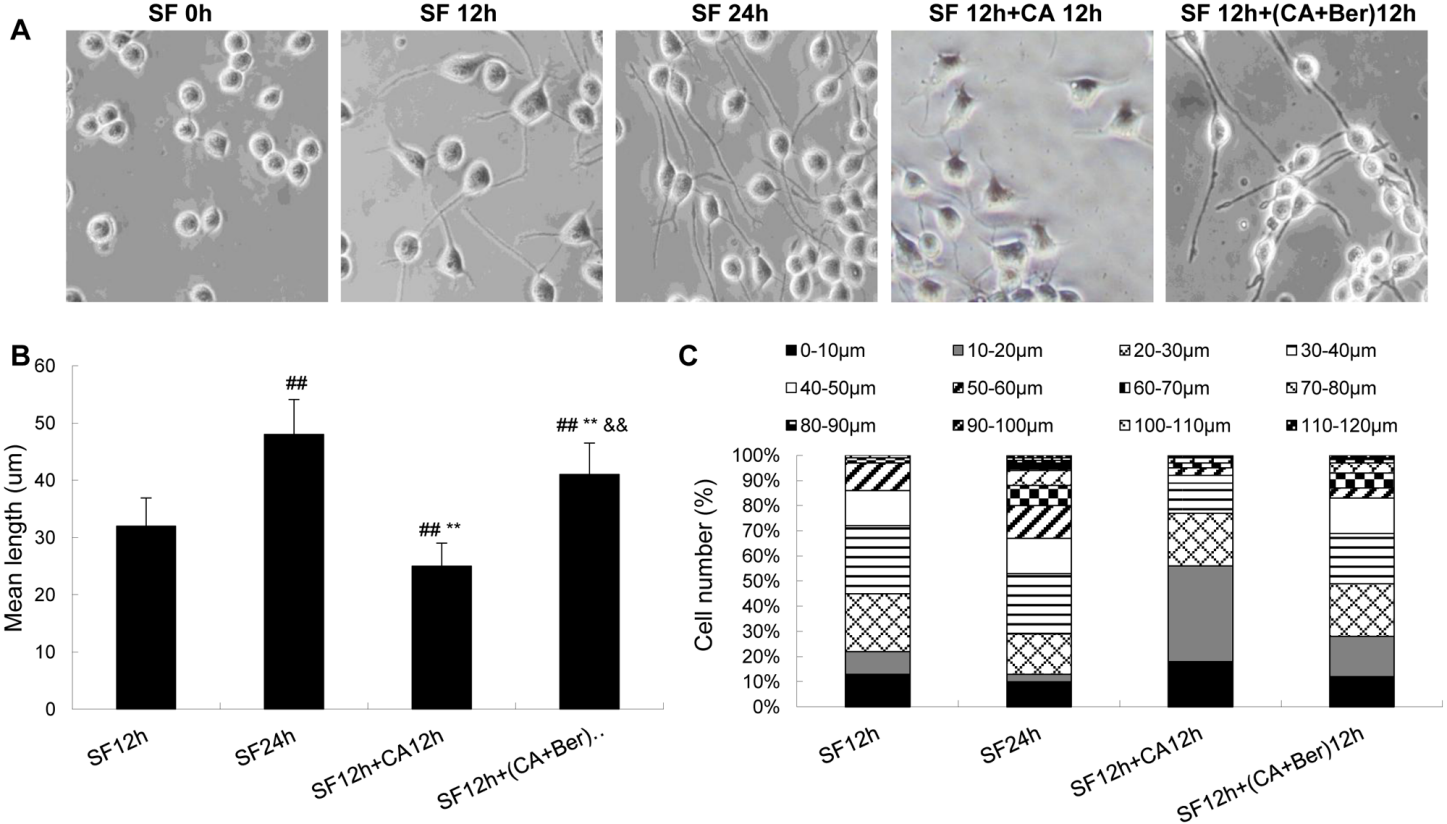
CA inhibits serum withdrawal-induced outgrowth of axon-like cell processes and protection by Ber in N2a cells. The cells were cultured in serum-free (SF) medium for 12 or 24 h, then 2.5 nmol/L CA was added to the 12 h SF group and these cells were cultured for another 12 h. The morphology of the cells was photographed by a phase contrast microscope (**A**). The mean length of the cell processes (**B**, excluding those with zero length of the processes) and the proportion of cells with different lengths of the processes (**C**) were calculated by a stereological system. About 100–200 axon-like cell processes for each treatment were counted. Data were mean ± SD (n  =  5). ^##^p<0.01 versus SF 12 h, **p<0.01 versus SF 24 h, ^&&^p<0.01 versus SF 12 h+ CA 12 h. Bar   =  10 μm.

## Supporting Information

S1 File Raw images used to create revised panels in Fig 5. (ZIP)
