# Supplementary figures and images for: Correction: Berberine Attenuates Axonal Transport Impairment and Axonopathy Induced by Calyculin A in N2a Cells
Source: PLoS One. 2016 Mar 24;11(3):e0152609. doi: 10.1371/journal.pone.0152609 (PMC4806838; doi:10.1371/journal.pone.0152609)

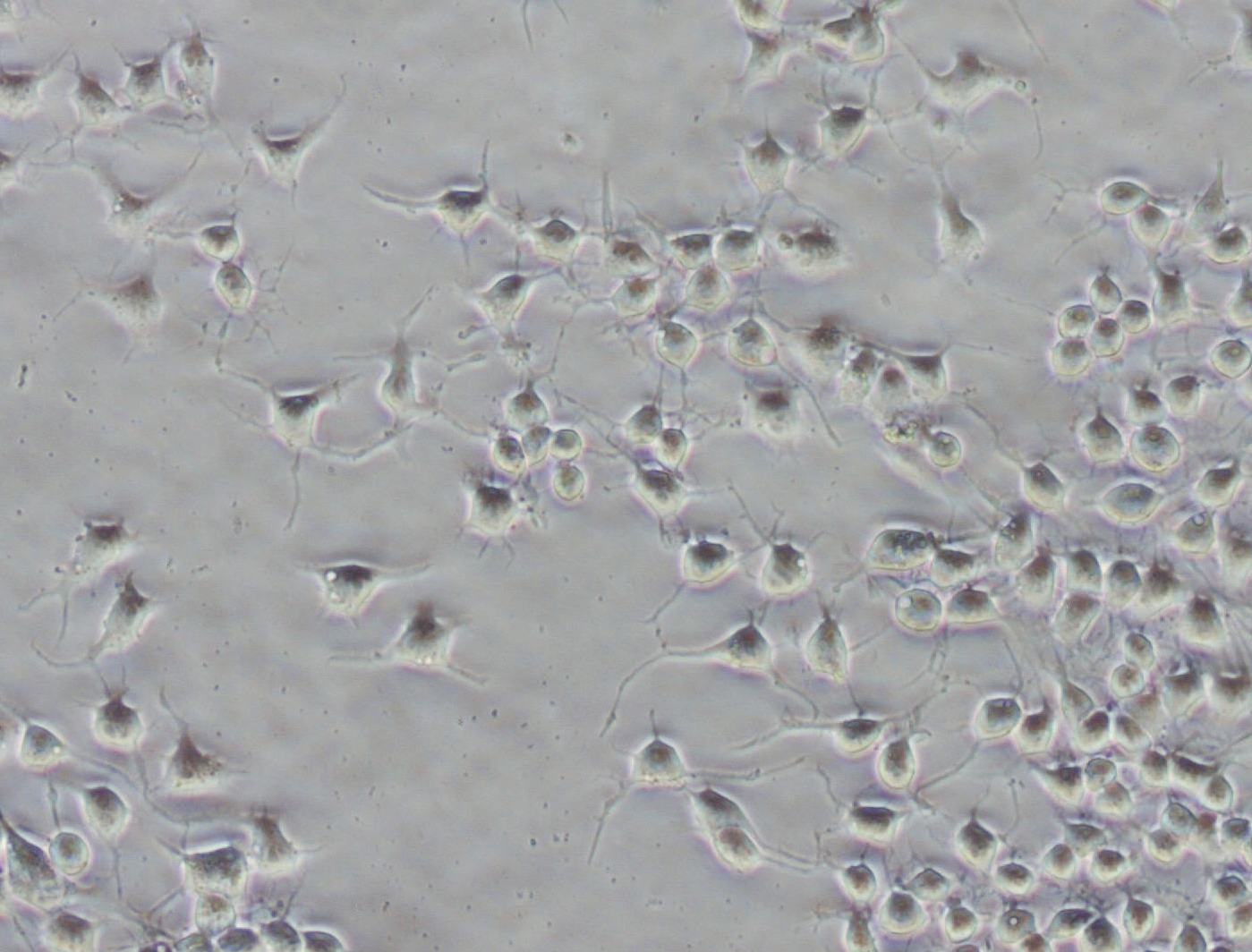

Supplement: S1 File — (ZIP) [file pone.0152609.s001.zip › Raw image of SF12h+CA12h.jpg]

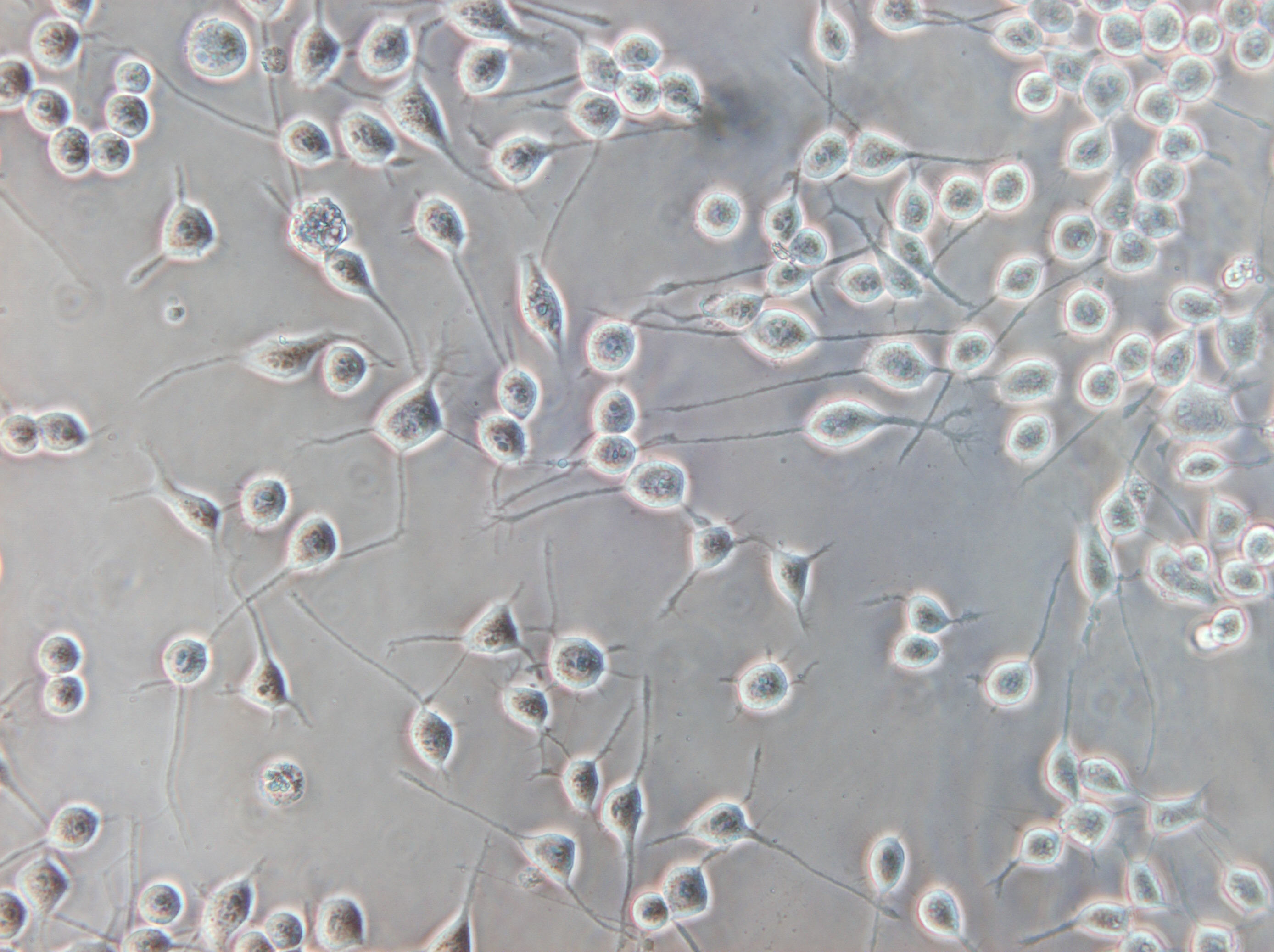

Supplement: S1 File — (ZIP) [file pone.0152609.s001.zip › Raw image of SF12h.jpg]

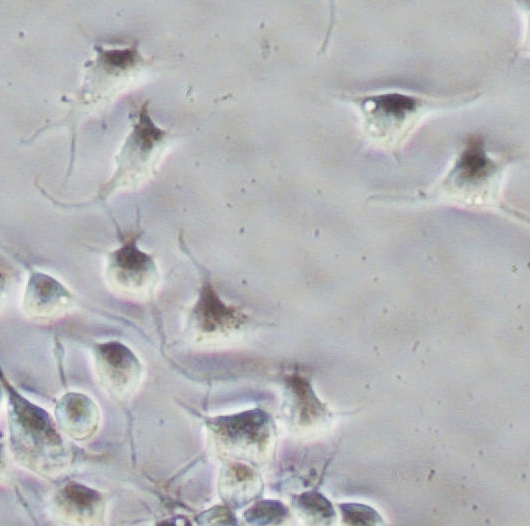

Supplement: S1 File — (ZIP) [file pone.0152609.s001.zip › SF12h+CA12h of new Figure 5.jpg]
